# Platelet-rich plasma versus corticosteroid injection for treatment of trigger finger: study protocol for a prospective randomized triple-blind placebo-controlled trial

**DOI:** 10.1186/s13063-020-04907-w

**Published:** 2020-11-27

**Authors:** Samuli Aspinen, Panu H. Nordback, Turkka Anttila, Susanna Stjernberg-Salmela, Jorma Ryhänen, Jussi Kosola

**Affiliations:** 1 Department of Hand Surgery, Helsinki University Hospital, Topeliuksenkatu 5, 00260 Helsinki, Finland; 2 Department of Orthopaedics and Traumatology, Kanta-Häme Central Hospital, Ahvenistontie 20, 13530 Hämeenlinna, Finland

**Keywords:** Clinical trial, Platelet-rich plasma, Trigger finger, Trigger thumb, Stenosing tenosynovitis

## Abstract

**Background:**

Trigger finger is a common hand disorder that limits finger range of motion and causes pain and snapping of the affected finger. Trigger finger is caused by an imbalance of the tendon sheath and the flexor tendon. The initial treatment is generally a local corticosteroid injection around the first annular (A1) pulley. However, it is not unusual that surgical release of the A1 pulley is required. Moreover, adverse events after local corticosteroid injection or operative treatment may occur. Platelet-rich plasma (PRP) has been shown to be safe and to reduce symptoms in different tendon pathologies, such as DeQuervain’s disease. However, the effects of PRP on trigger finger have not been studied. The aim of this single-center triple-blind randomized controlled trial is to study whether PRP is non-inferior to corticosteroid injection in treating trigger finger. The secondary outcome is to assess the safety and efficacy of PRP in comparison to placebo.

**Methods:**

The trial is designed as a randomized, controlled, patient-, investigator-, and outcome assessor-blinded, single-center, three-armed 1:1:1 non-inferiority trial. The patients with clinical symptoms of trigger finger will be randomly assigned to treatment with PRP, corticosteroid, or normal saline injection. The primary outcome is Patient-Rated Wrist Evaluation and symptom resolution. Secondary outcomes include Quick-Disabilities of the Arm, Shoulder and Hand; pain; grip strength; finger active range of motion; and complications. Appropriate statistical methods will be applied.

**Discussion:**

We present a novel RCT study design on the use of PRP for the treatment of trigger finger compared to corticosteroid and normal saline injection. The results of the trial will indicate if PRP is appropriate for the treatment of trigger finger.

**Trial registration:**

ClinicalTrials.gov NCT04167098. Registered on November 18, 2019.

## Background

Trigger finger (stenosing tenosynovitis, TF) is a condition of the tendons of the hand that causes triggering, snapping, or locking on flexion of the involved finger. TF can limit the range of motion of the affected finger and is frequently accompanied by pain in the palm of the hand [1]. TF is often considered under the category of “repetitive strain injury” [2]. The snapping of the finger is caused by the imbalance of the tendon sheath and the flexor tendon, or more precisely, thickening of the first annular pulley, the tendon, or both. Although known as tenosynovitis, no inflammatory changes have been observed in histologic studies [3]. While the condition has been recognized since 1850, the exact etiology of the condition remains unknown [4].

TF is a very common hand disorder with an incidence of approximately 3% in the general population [4]. The incidence increases to 10% among patients with diabetes [5]. Besides diabetes, gout, carpal tunnel syndrome, DeQuervain’s and Dupyutren’s disease, amyloidosis, and mucopolysaccharidosis are thought to be associated with TF [4]. TF occurs in women six times more frequently than men and is most common during the middle fifth to sixth decades of life. The ring finger is the most commonly affected finger [6].

Conservative treatment of TF is mainly based on corticosteroid injections around the tendon sheath, where it is hypothesized to decrease the tendon-sheath disproportion [7]. Nonsteroidal anti-inflammatory agents, massage, heat, ice, splinting, exercises, and stretches might also be considered as a conservative approach to relieve symptoms [8]. The short-term effect of a single corticosteroid injection has been evaluated to set between 60 and 92% [1], whereas the incidence of spontaneous recovery has been estimated to be as high as 20–29% [3]. There are three previously published randomized controlled studies comparing corticosteroid and saline injections for primary TF [9–11], advocating the use of corticosteroid injection. The short-term symptom resolution of corticosteroid versus saline injection in these studies was 54–64% versus 15–27%, respectively. While short-term results support the use of corticosteroid injection in primary TF, the efficacy seems to decrease over time and the long-term evidence is insufficient [12, 13].

If conservative treatment of TF fails or symptoms recur, and the patient is compliant, surgical release of the A1 pulley should be considered [14]. Surgical release of the pulley structure may be executed in an open, percutaneous, or endoscopic manner with uncertain comparative evidence; open release is the most used and traditional method [4].

Platelet-rich plasma (PRP) has been shown to reduce symptoms in different tendon pathologies [15, 16] and seems to be superior to cortisone [17–19]. PRP therapy is safe and feasible [20] but does not reverse the degenerative tendon changes [21]. On the other hand, cortisone treatment is not a long-term solution due to the relatively high recurrence rates after injections [22] and can cause adverse events in patients [23].

Considering the possible adverse events relating to open or percutaneous TF surgery [24], novel methods should also be considered. As PRP therapy seems to be safe and effective in many tendon pathologies, we designed a prospective placebo-controlled randomized trial to evaluate the possible benefits of PRP therapy in TF treatment. Our primary hypothesis is that PRP is non-inferior to cortisone measured with symptom resolution and Patient-Rated Wrist Evaluation (PRWE). The secondary hypothesis of this trial is that single injection of PRP is superior compared to placebo in the treatment of TF.

## Methods/design

### Trial hypothesis

Our primary hypothesis is that PRP is non-inferior to cortisone, and the secondary hypothesis of this trial is that single injection of PRP is superior compared to placebo in the treatment of TF. The primary endpoint is the Patient Rated Wrist Evaluation (PRWE) score and symptom resolution at 6 months post-injection. This will also be the timepoint for unveiling the primary allocation. Secondary objectives include Quick-Disabilities of the Arm, Shoulder and Hand (Q-DASH); pain (Visual Analogue Scale, VAS); grip strength; active range of motion (ROM); rate of adverse events; and global improvement.

### Trial design

**Fig. 1:**
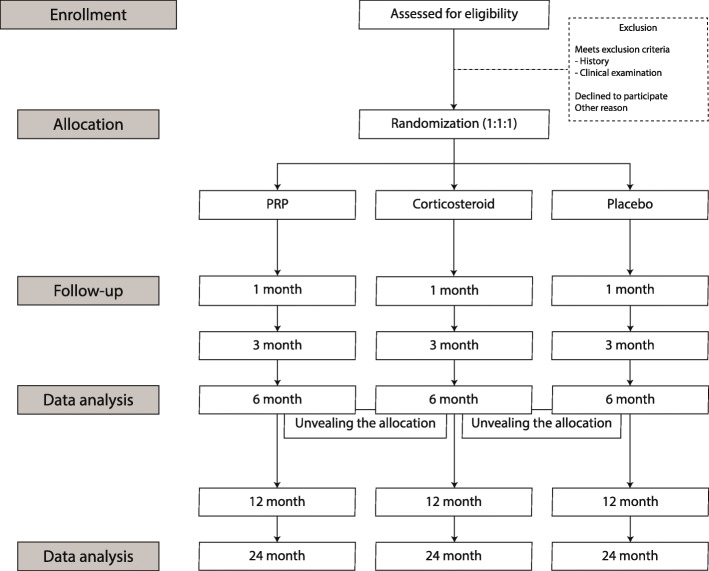
CONSORT diagram of the trial

The trial is designed as a randomized, controlled, patient-, investigator-, and outcome assessor-blinded, single-center, three-armed 1:1:1 non-inferiority trial. The CONSORT diagram of the trial cohort is presented in Fig. 1.

### Participant characteristics

We will assess the eligibility of all patients with TF that are referred to Helsinki University Hospital. These participants will be screened according to the inclusion and exclusion criteria. A recruitment investigator (RI) will confirm the clinical diagnosis of TF. The diagnosis is based on the following clinical findings: tenderness/pain over the flexor tendon sheath (A1 pulley), thickening or swelling of the tendon and/or the tendon sheath, and triggering or snapping of the affected finger. To qualify as a RI, all trial physicians must have experience of treating more than 200 cases of TF before the start of the trial.

Patients eligible for this trial will receive written information. The RI will obtain consent from the participants and collect baseline data prior to the randomization. The eligibility criteria are presented in Table 1. Participation in the trial is voluntary, and withdrawal is allowed at any time. In case of inadequate symptom relief after injection, the allocation will be unveiled. The patient will be offered either a corticosteroid injection (cross-over or second injection) or open surgery under local anesthesia, but preferably not before 6 months after the initial treatment.

**Table 1:** Trial eligibility criteria

Inclusion criteria (all of the following)	Exclusion criteria (any of the below)
Age 18–75	Diabetes
Trigger finger in 1–2 rays of the affected hand	Trigger finger in > 2 rays of affected hand
Symptom duration > 3 months	Rheumatoid arthritis or other condition requiring continuous oral corticosteroids
	Previous history of surgery or injection in the affected ray
	Dupuytren’s disease of the affected hand
	Alcohol or drug abuse
	Mental instability

### Preparation of PRP, corticosteroid, and placebo

PRP is prepared according to the manufacturer’s instructions (Regen Lab SA, Switzerland). Each voluntary patient eligible for the trial will have 10 ml of fresh venous blood withdrawn in a sterile manner into PRP tubes by a research nurse to ensure blinding of the treatment. The nurse will then move to another room, randomize the patient, and prepare the product indicated by the randomization. After centrifugation, 0.5 ml of ready-made PRP will be used. The corticosteroid group will receive 0.5 ml of Depo-medrol, and the control group will receive 0.5 ml of 0.9% saline. The syringe used will be taped by the nurse with sterile opaque tape and provided to the investigator. To ensure concealment, the research nurse will not participate further in the treatment or follow-up of the patients.

### Randomization and concealment

A randomization sequence will be generated by an independent investigator (JK) not involving in the execution of the trial using an internet-based program (sealedenvelope.com). Patients will be allocated to one of the three treatment groups in a 1:1:1 ratio using permuted block randomization with variable block size. The randomization will be performed by the research nurse by opening a sequentially numbered sealed opaque envelope after the RI has confirmed the eligibility and the voluntary participance of the patient. The envelopes will be kept in a secure, lockable cabinet that is only accessible by the study nurse. Neither the patient nor the RI will know the treatment product indicated by the randomization.

### Interventions

All injections will be administered by a trained hand surgeon (SS-S) or a hand surgeon in training (PN, SA, TA). Each of these physicians has the experience of treating more than 200 cases of TF before the start of the trial. The injection technique will be landmark based in each group.

Patients will be injected under aseptic conditions with a 23-gauge needle. During the injection, patients will be in a seated position with their wrists resting in supination on the table. The flexor tendon and the first annular pulley will be palpated. The needle will be introduced through the skin and into the supratendineous space at a slight oblique angle oriented distal to proximal. The patient will then be asked to flex and extend the finger; if the needle and the syringe hold still, the injection will be given. In this way, we will avoid accidental intratendinous injection.

Concomitant treatment with nonsteroidal anti-inflammatory agents, massage, heat, ice, exercises, and stretches are permitted, whereas repeated injections and splinting are prohibited.

### Outcome measures

**Fig. 2:**
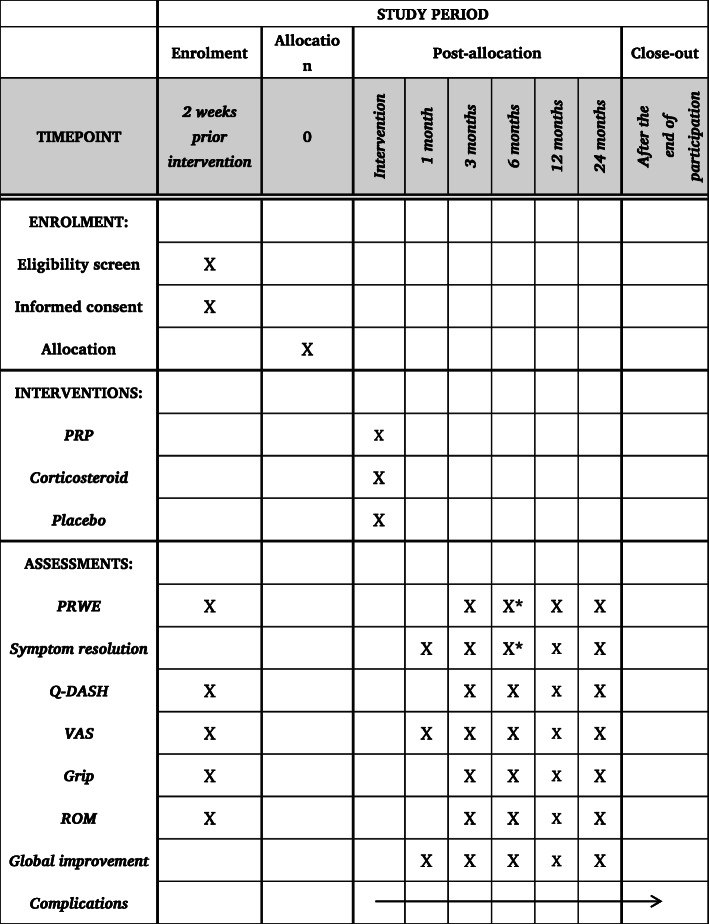
The schedule of enrolment, interventions, assessments, and data collection. PRP, platelet-rich plasma; PRWE, Patient-Rated Wrist Evaluation; Q-DASH, Quick-Disabilities of the Arm, Shoulder and Hand; ROM, finger active range of motion; VAS, Visual Analogue Scale. * = primary endpoint of the study

The primary enpoints are PRWE and symptom resolution at 6 months post-injection. Secondary outcomes include Q-DASH, pain on Visual Analogue Scale (VAS, 0 = no pain, 10 = worst imaginable pain), grip strength, finger active range of motion (ROM), and global improvement. The possible recurrence of TF symptoms will be registered. After initial treatment, follow-up will take place at 1, 3, 6, 12, and 24 months post-treatment. The trial schedule of enrolment, interventions, assessments, and data collection is presented in Fig. 2.

#### Patient-Rated Wrist Evaluation

The Patient-Rated Wrist Evaluation (PRWE) is a simple standardized outcome measure for wrist and hand pathologies that is easy to administer and score in clinical practice. The PRWE is a 15-item questionnaire designed to measure pain and disability in activities of daily living [25]. The Finnish version has been translated, culturally adapted, and validated [26].

#### Symptom resolution

Symptom resolution is a consensus between the patient and doctor on the perceived benefit of the treatment and is rated as follows: 0 = no response; 1 = partial response, but not satisfactory, warranting further treatment; 2 = partial response, satisfactory, not warranting further treatment; and 3 = complete resolution of symptoms and signs.

#### Quick-disabilities of the Arm, Shoulder and Hand

The Quick-Disabilities of the Arm, Shoulder and Hand (Q-DASH) is a 11-item questionnaire that considers the upper extremity as one functional unit [27]. It is a widely used reference for self-reported disability in various pathologies that affect the upper limb. Similar to the PRWE, the Finnish version has been translated, culturally adapted, and validated [28].

#### Pain

The Visual Analogue Scale (VAS) consists of a straight line with endpoints that define extreme limits to experiencing pain, from “no pain at all” and “pain as bad as it could be” [29]. The subject is asked to mark their pain level on the line between the two endpoints. The distance between “no pain at all” and the mark defines the subject’s pain. The VAS is a validated and reliable tool in pain assessment and is easy to use [30].

#### Grip strength and finger range of motion

**Fig. 3:**
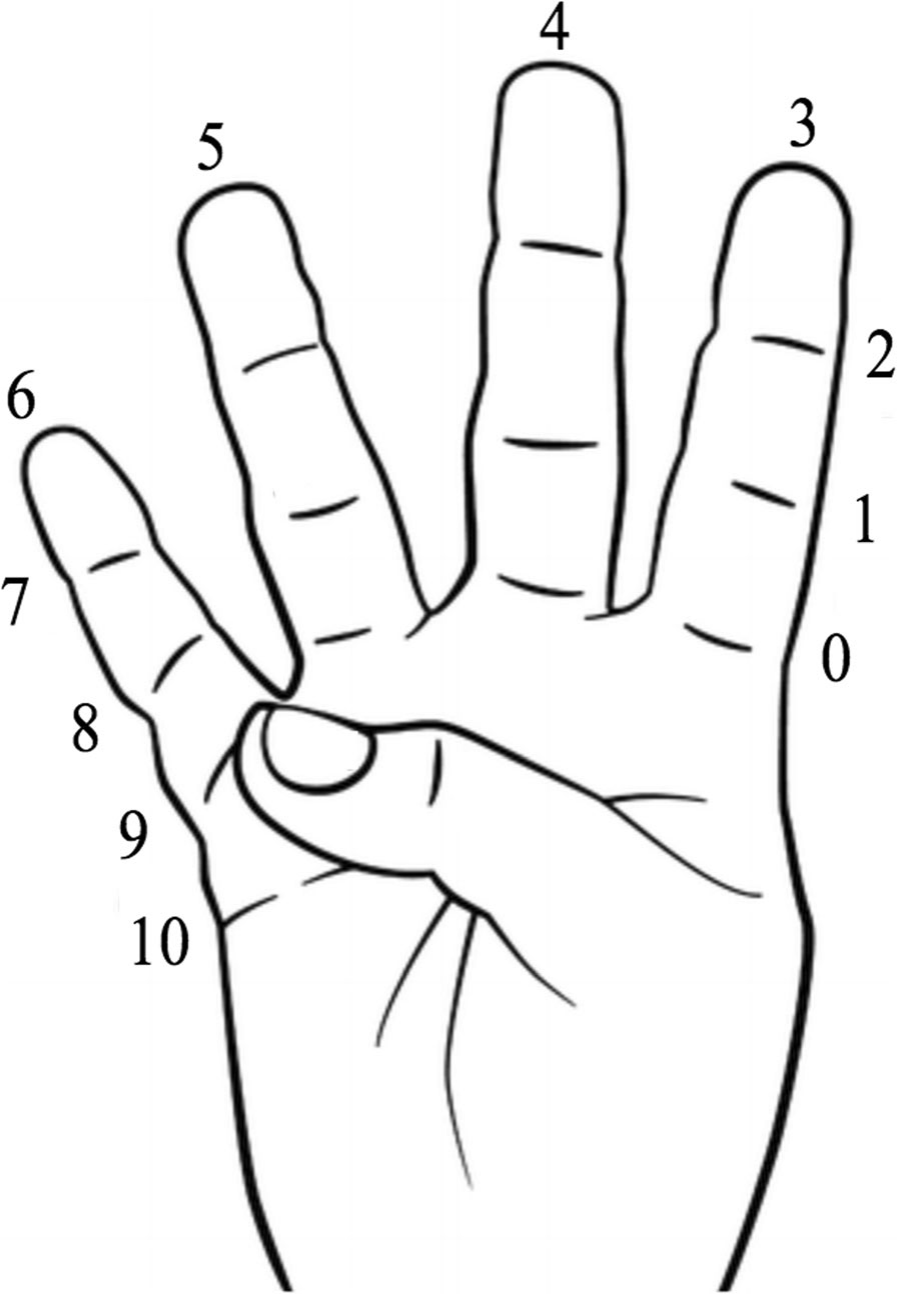
Kapandji thumb opposition score (0 = no opposition, 10 = maximal opposition)

Grip strength will be determined with a dynamometer (JAMAR hand dynamometer Model J00105, Lafayette, IN, 47903, USA). Finger active range of motion (ROM) will be measured using a manual goniometry. In addition to thumb interphalangeal joint goniometry, thumb active ROM is measured by using the Kapandji thumb opposition score (Fig. 3) [31].

#### Global improvement

Global improvement is a patient-centered standpoint of perceived benefit of the intervention. Global improvement is evaluated using five-step Likert scale from (− 2) “Much worse” to (+ 2) “Much better.”

#### Adverse events

Complications will be reported. Tendon, nerve, arterial injury, chronic regional pain syndrome, infection, hematoma, or any other condition that can be attributed to the intervention will be regarded as adverse events (event needing intervention or not disappearing).

### Data collection, management, and statistical plan

#### Data collection

All RIs will be trained for trial electronic database use, injection technique, ROM, and grip measurements. For PRWE and Q-DASH questionnaires, forms on paper will be the primary data collection tools. When receiving the questionnaire forms, a trial nurse will make a visual check of the responses and queries for missing data when possible. Furthermore, the trial nurses will be educated on preparation of PRP, corticosteroid, and placebo. RIs will be blinded to group allocation and will store the forms in an electronic database by double data entry to minimize typing errors. Patient records will be reviewed when collecting missing data or interpreting inconsistent or implausible data.

#### Data management and monitoring

A database including patients’ identification information and consent forms will be generated. This database will also include the identification code given to each patient and the intervention. Questionnaire forms on paper are the primary data collection tools for the study. As the questionnaire forms are received, a study nurse will make an inspection of the responses and inquire missing data when possible. Research assistant, blinded to the group allocation, will store the forms into a password-protected electronic database on a hospital-provided server by double data entry to minimize typing errors.

No separate data monitoring committee will be established. As collected outcome data is de-identified regarding trial group, the RIs and biostatistician can control for safety of the interventions during the trial without compromising concealment. An interim analysis of the de-identified data will be run after 40% of the patients are enrolled and followed for 6 months. If any concerns arise regarding trial safety, the concealment will be unveiled, and the trial will be discontinued.

To ensure correct execution of the study, audits may be conducted if deemed necessary. However, routine audits are not planned.

#### Sample size

The primary outcome measure is PRWE, and the primary hypothesis of our trial is that single injection of PRP is non-inferior to single injection of corticosteroid in the treatment of TF measured with PRWE total score. The non-inferiority margin is set at 11.5 points using the PRWE minimally clinically important difference [32, 33]. To exclude the non-inferiority margin, the trial will require 32 patients in each group to observe MCID (non-inferiority margin 11.5, SD 14) in PRWE scores between the trial groups with a power of 90% and using a one-sided type I error rate of 2.5%. We will recruit 117 patients to account for 20% loss during follow-up.

#### Statistical plan

Statistical analysis will be performed with an intention-to-treat method. A statistical software program will be used for analyzing entered data (IBM SPSS Statistics for Windows, Version 25.0. Armonk, NY: IBM Corp. 2017). Blinded data interpretation will be used to diminish interpretation bias; therefore, the biostatistician will be unaware of the group assignments when performing the analyses [34].

Descriptive statistics will be presented as mean (standard deviation) or median (interquartile range) for continuous variables and count (percent) for categorical variables. Levene’s test will be used to examine the homogeneity of variance between groups, and Welch’s or Student’s *t* test will be used to compare the point estimates for the means of the intervention groups. Ninety-five percent confidence intervals will be calculated for the mean difference. Chi-square test or Fisher’s exact test will be used to assess categorical data. Repeated measures will be analyzed using generalized linear mixed models.

The criterion for statistical significance will be set at *p* = 0.025 (one-sided) or *p* = 0.05 (two-sided). All *p* values will be reported to three decimal places with those less than 0.001 reported as *p* < 0.001.

### Cross-over, loss to follow-up, and missing data

Participants enrolled will be allowed to call for unveiling the concealment at any point of the trial (preferably no sooner than 6 months post-randomization). However, to minimize this, the following will be addressed prior to trial enrolment to ensure that potential participants:

* Are willing to receive any of the interventions

* Understand that treatments might not provide benefit

* Are willing to remain with their allocation for 6 months

In case the allocation is unveiled, the patient will be offered either a corticosteroid injection (cross-over or second injection) or open surgery under local anesthesia based on the patients’ preference.

We will document the number and proportion of patients eligible for and compliant with each follow-up. If the number of patients withdrawing from any arm of the trial is greater than the anticipated 20% at 6 months, an analysis of the demographic and prognostic characteristics will be performed between the individuals who withdraw and those who remain in the trial. Moreover, data may not be available due to voluntary withdrawal of patients, lack of completion of individual data items, or general loss to follow-up. Where possible, the reasons for missing data will be ascertained and reported. If judged appropriate, missing data will be imputed using MICE [35] and in concordance to the respective manuals of the questionnaires used. However, the main analysis will be done using the available (not the imputed) data.

### Regulatory aspects

Platelet-rich plasma is a biologic drug. However, it is not considered to be part of human cells, tissues, and cell- and tissue-based products (HCT/Ps, Title 21 United States Code of Federal Regulations Part 1271 (21 CFR 1271)). PRP is regulated by regulating the device used to manufacture it. In the case of PRP, the original predicate device is a platelet and plasma separator that produces PRP [36]. Likewise, Finnish Medicines Agency (FIMEA) does not consider PRP as a regulated drug, and thus, the approval for running this trial was not applied from FIMEA.

### Ethics and dissemination

Corticosteroid injections are widely used and considered safe and effective in the conservative treatment of TF. PRP is prepared from autologous blood and is inherently safe. Any concerns associated with allografts or xenografts regarding transmission of diseases, such as human immunodeficiency virus or hepatitis, or triggering of immunogenic reactions are eliminated [37]. The placebo consists of 0.9% saline and is safe and appropriate [9–11].

The trial will be conducted according to the Declaration of Helsinki. The study was approved by the Helsinki and Uusimaa Hospital District Ethical Committee (reference number HUS/2845/2019). The protocol is registered with ClinicalTrials.gov (trial identifying number NCT04167098, Table 2). The trial protocol was developed according to the Standard Protocol Items: Recommendations for Interventional Trials (SPIRIT) statement. The findings of this trial will be disseminated through peer-reviewed publications and conference presentations.

**Table 2:** Content of the trial registry

Data category	Information
Primary registry and trial identifying number	ClinicalTrials.gov; NCT04167098
Date of registration in primary registry	November 18, 2019
Date and version identifier	October 9, 2020, version 1.2
Source(s) of monetary or material support	–
Primary sponsor	Töölö Hospital
Secondary sponsor	University of Helsinki
Contact for public queries	Samuli Aspinen, +358406360546, samuli.aspinen@hus.fi
Contact for scientific queries	Samuli Aspinen, +358406360546, samuli.aspinen@hus.fi
Public title	Effectiveness of Platelet-rich Plasma for Treatment of Trigger Finger
Scientific title	A Prospective Randomized Placebo-controlled Trial Comparing Platelet-rich Plasma and Corticosteroid Injection for Treatment of Trigger Finger
Countries of recruitment	Finland
Health condition(s) or problem(s)	Tendon entrapment
Intervention(s)	(1) PRP injection, 0.5 ml platelet-rich plasma around A1 tendon sheath; (2) corticosteroid injection, 0.5 ml methylprednisolone around A1 tendon sheath; and (3) placebo injection, 0.5 ml 0.9% saline around A1 tendon sheath
Key inclusion and exclusion criteria	Inclusion: age 18–75, trigger finger in 1–2 rays, symptom duration > 3 months Exclusion: diabetes, rheumatoid arthritis or other condition requiring continuous oral corticosteroids, > 2 affected rays, previous history of surgery or injection to the affected ray, alcohol or drug abuse, mental instability
Study type	Interventional
Date of first enrolment	April 9, 2020
Target sample size	117
Recruitment status	Recruiting
Primary outcome(s)	Symptom resolution (rate of success), Patient-Rated Wrist Evaluation
Key secondary outcomes	Quick-Disabilities of the Arm, Shoulder and Hand; pain (Visual Analogue Scale); global improvement; grip strength; finger range of motion (ROM); complications

*PRP* platelet-rich plasma

## Discussion

An increasing interest in biologic agents as nonoperative treatment modalities or to augment surgical procedures has been observed in recent years. One of these, PRP, has been shown to reduce symptoms in different tendon pathologies with the rationale to potentially accelerate the healing process [38].

PRP has positive effects on both short-term and long-term pain on tendon and ligament healing [39]. PRP contains various growth factors that have potential tendon-healing properties [38]. PRP has been previously used in hand pathologies such as osteoarthrosis [40]. Furthermore, Ramesh et al. [41] reported a 77% success rate after one and a 93% success rate after two doses of autologous PRP for the treatment of DeQuervain’s disease. The effects of PRP on TF have not yet been studied.

Digital stenosing tenosynovitis (i.e., TF) is a common example of a tendon pathology caused by the imbalance of the flexor and its sheath. Injection of corticosteroids in the vicinity of the A1 pulley is generally accepted as a first-line therapy, although recurrence rates up to 33% have been reported [1]. Moreover, up to 5.8% of major adverse events have been reported in soft-tissue injections of cortisone (defined as those needing intervention or not disappearing) [23]. As some authors have stated superiority of PRP compared to cortisone in select musculoskeletal disorders [17–19], investigating the clinical efficacy of PRP in treating TF is warranted.

Despite the increasing interest and use of PRP in clinical orthopedics, there are still concerns regarding its clinical efficacy due the lack of high-quality randomized controlled trials. Contradictory results have also been observed. A participant-, investigator-, and assessor-blinded, placebo-controlled, randomized trial setting is the reference standard in trial designs. Thus, we conceived this trial to assess the effectiveness of PRP intervention in treating TF of the digits in a high-quality trial setting by measuring patient-rated outcomes, symptom reduction, and improvement of function. Moreover, to the best of our knowledge, this study is the first clinical trial to assess the use of PRP in TF.

## Limitations

The main limitation of the study will be the use of non-standardized PRP preparations. The number of platelets in a batch of PRP cannot be determined during the procedure, and controlling the concentration of platelets and leukocytes in a given batch of PRP is difficult [42]. Thus, we decided to use a pragmatic approach to PRP intervention that simulates the genuine clinical setting in which PRP is most commonly administered.

## Trial status

The recruitment phase of the trial has started. The first participant was randomly assigned on 9 April 2020. Recruitment is expected to be completed by December 2021. This protocol is version 1.2, dated 9 October 2020. Trial completion is expected by December 2023.

## Data Availability

The datasets generated and analyzed during this trial will be available from the corresponding author on reasonable request.

## Abbreviations

PRP: Platelet-rich plasma
PRWE: Patient-Rated Wrist Evaluation
Q-DASH: Quick-Disabilities of the Arm, Shoulder and Hand
RI: Recruitment investigator
ROM: Finger active range of motion
TF: Trigger finger
VAS: Visual Analogue Scale
